# Complete genome sequence of the ammonia-oxidizing archaeon *Nitrosopumilus zosterae* type strain NM25

**DOI:** 10.1128/mra.00253-26

**Published:** 2026-04-23

**Authors:** Tatsunori Nakagawa, Yuki Tsuchiya, Reiji Takahashi

**Affiliations:** 1 College of Bioresource Sciences, Nihon University 622935 , Fujisawa, Kanagawa, Japan; Nanchang University, Nanchang, Jiangxi, China

**Keywords:** complete genome, ammonia-oxidizing archaeon, coastal sediment, *Nitrosopumilus*

## Abstract

This work describes the complete genome sequence of a chemoautotrophic ammonia-oxidizing archaeon *Nitrosopumilus zosterae* NM25^T^. The assembled genome is composed of a circular chromosome with a genome size of 1,775,052 bp and a G+C content of 33.91%. Overall, 2,055 protein-coding genes, 43 tRNA genes, and 3 rRNA genes were predicted.

## ANNOUNCEMENT

An ammonia-oxidizing archaeon *Nitrosopumilus zosterae* NM25^T^ was isolated from coastal sediment (34° 39′ 37″N, 138° 58′ 50″E) in the eelgrass zone (1, 2). *Nitrosopumilus* is widely distributed in marine environments (1). A complete genome of assembled *N. zosterae* NM25^T^ chromosome has not been published because of repeat regions, although the draft genome assembly including 14 contigs (BioProject: PRJDB6801) was already reported (1). Here, we report the closed genome sequence of *N. zosterae* NM25^T^ using PacBio high-fidelity (HiFi) reads.

**Fig 1:**
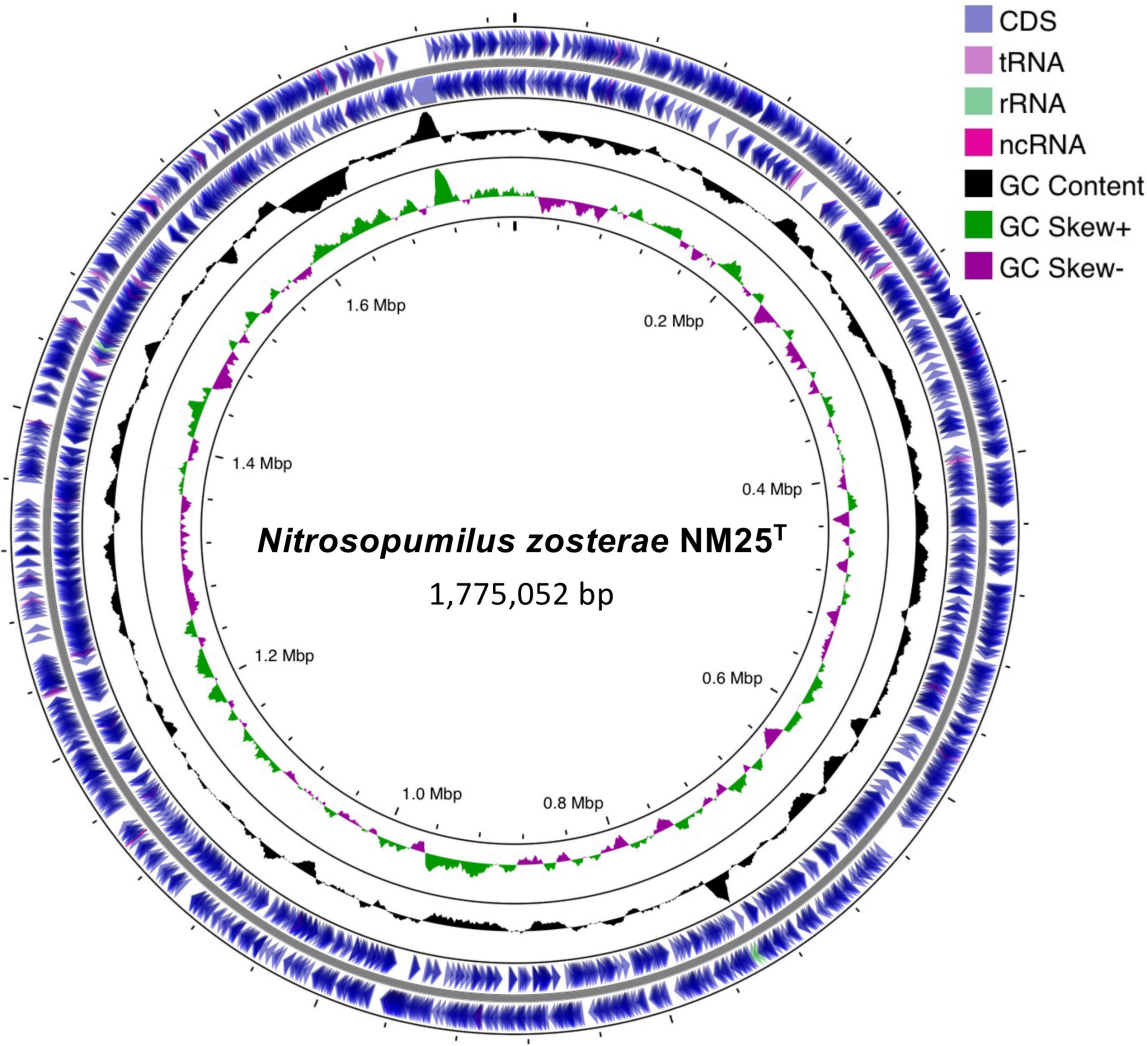
Circular genome map of *Nitrosopumilus zosterae* NM25^T^ visualized using Proksee (https://proksee.ca). The outer circular maps (blue) show the coding sequences (CDS), tRNA, rRNA, and noncoding RNA (ncRNA). The black circle represents the GC content. The inner circles (green and purple) represent the GC skew values, with green representing a positive skew and red representing a negative skew.

*N. zosterae* NM25^T^ was grown in a 10 L bottle closed with a screw cap, containing 4 L of HEPES-buffered SCM medium (2), at 30°C in the dark with stirring for 12 days. The cells were filtrated with a Millipore Stericap Vacuum Filter. The filter was added into a 50 mL plastic tube and stored at −20°C. The nucleic acids were extracted using a NucleoBond Buffer Set III and an AXG 500 column (TaKaRa Bio, Shiga, Japan) and purified using an AMPure XP beads (Beckman Coulter, CA, USA). The quality and quantity of the DNA were checked using an Agilent HS Genomic DNA 50 kb Kit (Agilent Technologies, CA, USA) and a QuantiFluor dsDNA System (Promega, WI, USA), respectively. The genome of the strain was sequenced on a PacBio Sequel IIe platform (Pacific Biosciences, CA, USA) using the Sequel II binding kit 2.2 (Pacific Biosciences) and the circular consensus sequencing (CCS) application. For the preparation of 10–20 kb fragments, 5 µg of genomic DNA was sheared using a g-TUBE (Covaris, MA, USA) at 2,100 × *g* in a microcentrifuge. The PacBio library was prepared using a SMRTbell Express Template Prep Kit 2.0 (Pacific Biosciences). An adapter was extracted from the reads using SMRT Link ver. 10.1.0.119,528 (Pacific Biosciences), resulting in 22,105 of HiFi reads covering 193,617,728 sequenced bases with a read N50 of 8,759 bp. The quality of the HiFi reads was assessed using Filtlong ver. 0.2.0 (https://github.com/rrwick/Filtlong) with “--min_length 1000” and “--keep_percent 90” parameters, resulting in 19,847 of HiFi reads. The filtered HiFi reads were assembled using Flye ver. 2.8.3 (3) with “--pacbio-hifi” and “-i 3” parameters and resulted in one circularized contig of 109× coverage and a total genome size of 1,775,052 bp with a G+C content of 33.91%. The *N*_50_ contig and the longest contig sizes were 1,775,052 bp. An assembly graph was constructed with Bandage v.0.8.1 (4) (default parameter), showing a single circular chromosome (1,775,052 bp). Genome completeness and contamination were estimated using CheckM (5) on the DFAST_QC pipeline ver. 1.0.7 (6) gene set with the “Thaumarchaeota” marker set, showing that the completeness was 98.49% and the contamination was 2.47%. Subsequently, the assembled genome sequence was annotated with NCBI Prokaryotic Genome Annotation Pipeline (PGAP) ver. 2022-04-14.build6021 (7), which resulted in 2,061 coding sequences (CDSs) with a single copy of the 5S, 16S, and 23S rRNA genes, 43 of tRNA genes, and two of noncoding RNA genes in the chromosome. The assembled genome was visualized as a circular map with Proksee ver. 2.0.3 (8), as shown in Fig. 1.

## Data Availability

The complete genome sequence of *N*. *zosterae* NM25^T^ has been deposited in GenBank/ENA/DDBJ with the accession number AP026695 under the BioProject accession number PRJDB14188, and PacBio raw reads have been deposited under SRA accession number DRX383787.
